# Non-linear GABA_A_ receptors promote synaptic inhibition in developing neurons: Commentary on Lodge et al. (2021) Sparsification of AP firing in adult-born hippocampal granule cells via voltage-dependent α5-GABA_A_ receptors. Cell Rep 37:109768

**DOI:** 10.1007/s00424-021-02652-w

**Published:** 2021-12-14

**Authors:** Knut Kirmse

**Affiliations:** Department of Neurophysiology, Institute of Physiology, University of Würzburg, 97070 Würzburg, Germany

Synaptic inhibition is chiefly mediated by GABA_A_ receptors (GABA_A_Rs). GABA_A_Rs form pentameric anion channels that typically assemble from two α, two β, and one tertiary (γ, δ, ε, θ, π, ρ) subunit, with different subunit combinations conferring different biophysical properties. GABA_A_R-mediated inhibition of action potential (AP) firing results from hyperpolarization and/or an increase in membrane conductance shunting excitatory inputs. Therefore, to ensure efficient inhibition, most mature neurons maintain a low chloride concentration ([Cl^−^]_i_). In contrast, immature neurons accumulate chloride [12], thus favoring depolarizing GABA responses in vivo [7, 14]. Whether excitatory or inhibitory actions of depolarizing GABA predominate is difficult to predict. This is due to complex interactions between the membrane (*V*_m_) and chloride reversal potentials, the GABA_A_R conductance, and the timing and location of synaptic inputs [4, 6, 8, 10]. However, in vivo studies confirmed that depolarizing GABA can effectively inhibit AP firing already in developing neurons [7, 13]. Although these observations suggest that immature neurons can prevent excess excitation through depolarizing GABA, the underlying mechanisms are not fully understood.

In their elegant recent study, Lodge and colleagues [9] discovered a crucial role for α5 subunit-containing GABA_A_Rs (α5-GABA_A_Rs) in this process. Focusing on adult-born hippocampal granule cells (GCs), the authors analyzed GABAergic inputs mediated by soma-targeting parvalbumin (PV) and dendrite-targeting somatostatin (SOM) interneurons. In acute hippocampal slices, in which young GCs are depolarized by GABA [4], both somatic and dendritic synapses were found to use similar GABA_A_Rs with a striking non-linear voltage-dependence, pharmacologically identified as α5-GABA_A_Rs. Their conductance at depolarized potentials close to AP threshold (− 35 mV) was fourfold larger than at resting *V*_m_ (− 80 mV). Previous work demonstrated that α5-GABA_A_Rs are high-affinity, slowly desensitizing receptors that can dynamically redistribute between extrasynaptic and synaptic sites in an activity-dependent manner [2, 3], thus mediating both tonic [5] and phasic [11] inhibition.

What are the functional consequences of outwardly rectifying α5-GABA_A_Rs? It has long been known that, under certain conditions, depolarizing GABA can enhance the activation of NMDA receptors (NMDARs) and might also facilitate AP firing. This could be of developmental importance, since NMDARs are required for several forms of synaptic plasticity, such as synapse unsilencing [1]. However, as the number of active GABAergic synapses increases, facilitation shifts to inhibition due to increased shunting of glutamatergic currents. Using a rigorous electrophysiological approach in combination with detailed biophysical modeling, the authors demonstrated that outward rectification of α5-GABA_A_Rs results in a dominance of the shunting effect at considerably lower levels of GABAergic activity. Consequently, voltage-dependent α5-GABA_A_Rs inhibit NMDAR-dependent currents and AP firing in a broader range of GABAergic activity. Collectively, the voltage-dependent increase in conductance of α5-GABA_A_Rs around AP threshold is crucial for preventing excess synaptic excitation in young GCs, when GABA is depolarizing.

Comparing the properties of GABAergic synapses in young with those in mature GCs, the authors discovered an intriguing example of developmental regulation. Whereas SOM-GC synapses utilize non-linear α5-GABA_A_Rs throughout development, α5-GABA_A_Rs become developmentally excluded from PV-GC contacts. Thus, as steady-state [Cl^−^]_i_ declines during development [4], PV interneurons transition to targeting linear GABA_A_Rs lacking α5 subunits. These results extend previous work indicating that, during the circuit integration of GCs, α5-GABA_A_Rs redistribute from synaptic to extrasynaptic sites, which strengthens their contribution to tonic inhibition [2].

The study by Lodge and colleagues [9] raises exciting questions. (1) What is the impact of non-linear α5-GABA_A_Rs in dendritic synapses of mature neurons? The authors’ modeling results indicate that both linear and non-linear GABA_A_Rs can mediate effective inhibition if steady-state [Cl^−^]_i_ is low. However, this analysis did not consider activity-dependent chloride loads, which could cause substantial [Cl^−^]_i_ shifts, especially in dendritic compartments (due to their high surface-to-volume ratio). (2) How is the abundance of outwardly rectifying vs. linear GABA_A_Rs regulated? Such a regulation may represent a powerful mechanism by which (developing) neurons can tune inhibition, independent of changes in [Cl^−^]_i_. (3) Does the use of outwardly rectifying α5-GABA_A_Rs reflect a general principle by which developing neurons prevent excessive excitation? Since α5-GABA_A_Rs inhibit spontaneous cortical network activity in neonatal mice in vivo [7], this seems plausible. In sum, by showing that non-linear α5-GABA_A_Rs promote synaptic inhibition with depolarizing GABA, Lodge and colleagues [9] bring us one step closer to understanding how developing neurons prevent runaway excitation during synaptogenesis and circuit integration.
